# Imaging the functional connectivity of the Periaqueductal Gray during genuine and sham electroacupuncture treatment

**DOI:** 10.1186/1744-8069-6-80

**Published:** 2010-11-16

**Authors:** Carolyn E Zyloney, Karin Jensen, Ginger Polich, Rita E Loiotile, Alexandra Cheetham, Peter S LaViolette, Peichi Tu, Ted J Kaptchuk, Randy L Gollub, Jian Kong

**Affiliations:** 1 Department of Psychiatry, Massachusetts General Hospital, Charlestown, MA, USA; 2 MGH/Massachusetts Institute of Technology/Harvard Medical School (HMS) Athinoula A. Martinos Center for Biomedical Imaging, Charlestown, MA, USA; 3 Osher Research Center, Harvard Medical School, MA, USA

## Abstract

**Background:**

Electroacupuncture (EA) is currently one of the most popular acupuncture modalities. However, the continuous stimulation characteristic of EA treatment presents challenges to the use of conventional functional Magnetic Resonance Imaging (fMRI) approaches for the investigation of neural mechanisms mediating treatment response because of the requirement for brief and intermittent stimuli in event related or block designed task paradigms. A relatively new analysis method, functional connectivity fMRI (fcMRI), has great potential for studying continuous treatment modalities such as EA. In a previous study, we found that, compared with sham acupuncture, EA can significantly reduce Periaqueductal Gray (PAG) activity when subsequently evoked by experimental pain. Given the PAG's important role in mediating acupuncture analgesia, in this study we investigated functional connectivity with the area of the PAG we previously identified and how that connectivity was affected by genuine and sham EA.

**Results:**

Forty-eight subjects, who were randomly assigned to receive either genuine or sham EA paired with either a high or low expectancy manipulation, completed the study. Direct comparison of each treatment mode's functional connectivity revealed: significantly greater connectivity between the PAG, left posterior cingulate cortex (PCC), and precuneus for the contrast of genuine minus sham; significantly greater connectivity between the PAG and right anterior insula for the contrast of sham minus genuine; no significant differences in connectivity between different contrasts of the two expectancy levels.

**Conclusions:**

Our findings indicate the intrinsic functional connectivity changes among key brain regions in the pain matrix and default mode network during genuine EA compared with sham EA. We speculate that continuous genuine EA stimulation can modify the coupling of spontaneous activity in brain regions that play a role in modulating pain perception.

## Background

Acupuncture has been used to alleviate pain for thousands of years. Multiple randomized controlled trials (RCTs) and many meta-analyses have concluded that acupuncture effectively relieves clinical pain for disorders such as knee osteoarthritis [1], migraine [2] and chronic low back pain [3-5]. For instance, three recent large trials found that acupuncture is statistically, clinically [3-5], and cost-effectively [6-10] superior to either optimal guidelines-based conventional therapy, or waitlist controls for chronic low back pain. However, the lack of a significant clinical difference between genuine or sham (placebo) acupuncture has raised skepticism and limited acupuncture's acceptance.

In parallel, brain imaging tools such as positron emission tomography (PET) and functional Magnetic Resonance Imaging (fMRI) have been used to investigate the neural mechanisms underlying acupuncture needle stimulation [11-28]} and acupuncture treatment effects [17,29-32]. The goal of these studies is to elucidate the changes in neural activity in brain networks associated with acupuncture needle stimulation and to link them to the therapeutic effects of treatment. The block- and event-related paradigms often used in fMRI studies, while well suited to studying the effects of brief intermittent acupuncture needle stimuli, are not suitable for studying some modes of clinical acupuncture treatment. For instance, one of the most important acupuncture treatment modalities, electroacupuncture (EA), is typically administered with continual stimulation for twenty minutes or more. Elucidating the neural substrates of long-duration EA stimulation requires a new approach.

Recently, functional connectivity MRI (fcMRI) has attracted the attention of brain imaging investigators [33-35]. This technique provides a potential fMRI approach appropriate for the study of EA. Based on the premise that low-frequency components of the spontaneous MR imaging signal can provide information about the intrinsic functional and anatomical organization of the brain [36,37], fcMRI investigates the connectivity between all brain regions and a specifically defined "seed", or region of interest (ROI). Recent findings using fcMRI have significantly enhanced our understanding of the intrinsic functional and anatomical connections of the brain, including those that underlie pain perception [38] and modulation [39,40], and pathological changes in these connections that are associated with chronic pain in patients [41-43]. Recently, investigators have also reported functional connectivity changes within the default mode network [44,45], sensorimotor networks [44], and amygdala-associated brain networks [46,47] after acupuncture needle stimulation, suggesting that this approach is sufficiently sensitive to detect neural modulation associated with acupuncture treatment.

Therefore, in this new analysis of previously unpublished data collected in a randomized placebo-controlled study [29,30], we investigated the functional connectivity of PAG changes during EA stimulation and sham EA stimulation at acupoints Large Intestine 3 and 4 (LI3 and LI4) on the right hand. The PAG was chosen for analysis because: 1) it is well known that the PAG plays a role in pain modulation [48-52]; 2) PAG has been found to play an important role in acupuncture analgesia [53-55]; and 3) in a previous analysis conducted using this same data set, we found that fMRI signal change to calibrated heat pain was inhibited at the PAG after genuine acupuncture treatment, but was not influenced by level of analgesia expectancy [30].

## Methods

This study utilized a previously collected fMRI dataset investigating brain mechanisms underlying genuine and sham electroacupuncture's analgesic effects. This paper will focus on the fMRI data collected during the acupuncture administration. Results on the treatments' analgesic effects - as measured by changes in subjective pain ratings and in objective brain response - have been published separately [29,30]. Please see the original papers for these results as well as for additional details on experimental procedures not relevant to the present manuscript.

### Subjects

Seventy-seven healthy, right-handed subjects participated in this study; all subjects were acupuncture naive and had no history of neurological or psychiatric disorders. As approved by the Massachusetts General Hospital's Institutional Review Board, all subjects provided their written consent to participate. Subjects were then randomized to one of four groups: genuine EA or sham EA paired with either high expectancy (HE) or low expectancy (LE) manipulation. After their participation, subjects were debriefed as to the goals of the experiment and un-blinded to their randomization.

### Experimental Protocol

This study consisted of two behavioral testing sessions and one fMRI scanning session, each separated by a minimum of three days. The goals of the behavioral sessions were to familiarize subjects with the heat pain rating system and to manipulate their expectations of acupuncture's analgesic effects. In the subsequent fMRI session, the brain networks involved in genuine and sham EA, as well as in HE and LE conditions, were examined. A general overview of the experimental design is presented below.

### Session 1

This first behavioral session was used to determine appropriate stimulus intensities of heat pain and to familiarize subjects with the pain rating scales. Briefly, temperatures eliciting subjective intensity ratings in the LOW pain range (~5; which indicates weak on the 0-20 Sensory Scale) and HIGH pain range (~15; strong) were selected for each individual using an ascending series of noxious stimuli (increasing by 1°C per stimulus). We then applied a series of 8 noxious stimuli, 4 HIGH pain and 4 LOW pain, presented in random order, and a series of 6 identical HIGH pain noxious stimuli to the right arm. Temperatures were adjusted when necessary to ensure that each subject's subjective ratings of HIGH and LOW remained in the desired range and the final temperature settings were used in the following sessions.

### Session 2

To prepare subjects for the expectancy manipulation, and in order to establish subjects' expectancy for the duration of the study, subjects were first told that responses to acupuncture can be variable and that a given subject's response tends to remain consistent across sessions. Subjects then viewed a Traditional Chinese Medicine meridian diagram and were falsely told that, according to previous literature, acupuncture could only produce analgesia on the meridian side of the arm.

Following the preparatory disclaimer, pain stimuli were applied according to the same procedures as session 1. Then, according to their randomization, subjects received either genuine or sham EA. Finally, subjects received post-treatment pain stimuli. Though all subjects were told they were receiving the same pre-treatment heat stimuli so as to test acupuncture's analgesic effect, we actually only followed this protocol on subjects in the LE groups. In the HE groups, we surreptitiously decreased temperatures on the side of the arm subjects were told was the treated meridian in order to elicit reduced pain ratings and give subjects an unmistakable experience of analgesia. (This expectancy manipulation is a modification from previous studies [39,56-61].)

### Session 3

Subjects were told that the procedures from Session 2 would be repeated inside the fMRI scanner. First, fMRI scans were acquired during the pre-treatment application of noxious thermal stimuli. Then, another fMRI scan was acquired during the administration of sham or genuine EA, which provided the data for the functional connectivity analysis described in this manuscript. Lastly, subjects were scanned post-acupuncture treatment while additional heat stimuli were applied. LE groups received the same temperature thermal stimuli as they had before treatment. Although the HE groups were informed that Session 2 procedures would be repeated, only one reduced temperature heat stimuli was administered on the meridian side of the right forearm arm to remind subjects of the analgesia they experienced in Session 2. All other heat stimuli, applied after this "expectancy boost," were delivered at their original temperatures in order to test the treatment effect. In this study, we will focus only on the electro-acupuncture stimulation scan; additional details on the results of the fMRI scans acquired during heat pain application are reported in our previous publications [29,30].

### Acupuncture administration

Identical genuine or sham EA was performed by a licensed acupuncturist at acupoints LI3 and LI4 on the right hand during Sessions 2 and 3. LI 3 is located in the depression proximal to the metacarpo-phalangeal joint. LI 4 is located on the dorsum of the hand, between the 1st and 2nd metacarpal bones, in the middle of the 2nd metacarpal bone on the radial side. Both LI 3 and LI 4 can produce analgesic effects according to acupuncture literature [62,63].

**Figure 1:**
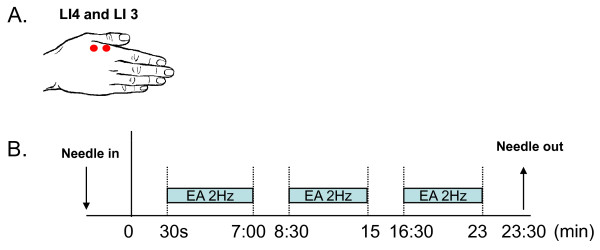
**Acupuncture stimulation procedures**. A) Anatomical location of the acupoints LI3 and LI4 on the right hand; used for genuine and sham acupuncture administration. B) Schematic illustration of the timing during treatment blocks. In total, electroacupuncture (EA) treatment was applied for approximately 25 minutes at a frequency of 2 Hz. Treatment was given in blocks of 6.5-minute current ON and four 1.5-minute current OFF blocks. For sham EA, the sham needle was attached to a de-activated EA device, and no current was applied during the 25 minute treatment.

For genuine EA, needles were inserted into the skin at a depth of about 1.5 cm and adjusted until subjective *deqi *sensations [64], but no sharp pain, was evoked. Needles were then connected to an EA device passing a 2 Hz current (OMS Medical Supplies IC-1107) [63], and the intensity was gradually increased to the highest level subjects could tolerate without the sensation of sharp pain. Once the appropriate level of stimulation was achieved for each subject, the level of current was recorded. After calibrating the intensity of the EA stimulation, treatment was applied for approximately 25 minutes. The treatment was further broken down into three 6.5-minute current ON and four 1.5-minute current OFF blocks. An fMRI scan was acquired for 23.5 minutes encompassing the entire treatment (Figure 1).

For sham EA, specially-designed Streitberger sham acupuncture needles were placed on the surface of the skin and connected to a de-activated electroacupuncture device. The Streitberger placebo needle has been validated and used in many studies [60,63,65-68]. Total scan time was the same for the sham group as for the genuine EA group.

After treatments, sensations evoked by genuine and sham EA were measured with the Massachusetts General Hospital (MGH) Acupuncture Sensation Scale (MASS), a rubric created by acupuncture researchers at MGH [63,64].

### fMRI Data Acquisition and Analysis

Brain imaging was performed with a 3-axis gradient head coil in a 3 Tesla Siemens MRI System equipped for echo planar imaging. At the midpoint of the study, an MRI scanner upgrade replaced the 3 Tesla head-only Siemens Allegra MRI System with a 3 Tesla whole-body Siemens TIM Trio MRI System. To avoid any potential confounding due to scanner hardware and software changes, pre-post scanner upgrade studies were conducted at the Martinos Center at the time of transition from the Allegra to the Trio scanner system and the data were used to optimize the image acquisition parameters to be as closely matched as possible while still taking advantage of the benefits of the scanner upgrade. Multiple previous studies have demonstrated that when these careful measures are taken to match scan acquisition parameters, inter-subject variability is the predominant source of variance in structural and functional MRI data, regardless of scanner type or manufacturer [69-73]. Therefore we were careful to randomize comparable numbers of subjects from each of the four groups across the two scanner systems: 6-8 subjects per group were tested on the old scanner and 4-6 subjects per group were tested on the new scanner.

Thirty axial slices (4 mm thick with 1 mm skip) parallel to the anterior and posterior commissure covering the whole brain were imaged with 2000 ms TR, 40 ms TE, 90° flip angle and 3.13 × 3.13 mm in-plane spatial resolution. For the scan acquired during EA stimulation, a total of 705 time points were collected during an acupuncture treatment lasting twenty-three minutes and 30 seconds. A high-resolution 3 D MPRAGE sequence for anatomic localization was also collected.

### Functional Connectivity Analysis

We used a spherical seed region located in the PAG, centered at 0 -28 -10 (Montreal Neurological Institute [*MNI*] coordinates) with a 2 mm radius. This seed served as a pre-defined anatomical region from which we explored connectivity to other brain regions. The seed region coordinates are based on our previous analysis of the fMRI signal changes evoked by calibrated heat pain before and after treatment using this same data set. The PAG voxel with the maximal pain-evoked fMRI signal change after genuine EA treatment, as compared with sham EA treatment, was located at 0 -28 -10 [30]. This location is situated in the ventral PAG, and as such, it belongs to the "analgesic zone" of the PAG [74].

Methods for functional connectivity analysis were similar to previous studies [75-77]. In summary, the long EA scan for each subject was split into 3 epochs (as illustrated in Figure 1) in which the current was turned on for participants receiving genuine EA, with each ON epoch lasting 6 minutes. (The first 30 seconds of each EA treatment was excluded to account for the time subjects spent acclimating to the application of the current from the EA device). Functional data were first preprocessed to decrease image artifacts and to eliminate differences in odd/even slice intensity. Rigid body translation and rotation was used to reduce within- and across-run head movement. Data were re-sampled to 3 mm isotropic voxels after transforming anatomical and functional data to atlas space.

The functional connectivity analysis required additional filtering of low- and high-frequency components (0.009 Hz < f < 0.083 Hz) and spatial 8 mm Gaussian kernel smoothing. Other variables that were simultaneously regressed included movement parameters, whole brain signal, lateral ventricle mean signals, deep white matter ROI signal, and the first temporal derivative of each time course. The resulting time course was used in the subsequent analysis. Next, we performed correlation maps between the seed region and all voxels across the whole brain. Analysis produced seed region-whole brain voxel correlation coefficients. Fisher's r-to-z transformation was used to convert correlation maps into z maps.

Group analysis was applied with a random effects analysis using a one sample t-test. To further explore differences in the functional connectivity of PAG between the different acupuncture modes and expectancy levels, an analysis equivalent to an ANOVA was performed. More specifically, to calculate the main effect of acupuncture, a two-sample t-test comparing the pre- minus post difference was performed between the cohort receiving genuine acupuncture treatment (pooling the two expectancy groups) and the cohort receiving sham EA treatment (pooling the two expectancy groups). Then, to calculate the main effect of expectancy, a two-sample t-test comparing the pre- minus post difference was performed between the high expectancy condition (pooling the acupuncture groups) and the low expectancy condition (pooling the acupuncture groups). Finally, to calculate the interaction between expectancy and acupuncture, a two sample t-test comparing the pre- minus post difference was performed between the cohorts receiving genuine EA with high expectancy + sham EA with low expectancy and the cohorts receiving sham EA with high expectancy + genuine EA with low expectancy. This method has been used in our previous studies [30] and is equivalent to the information provided by an ANOVA analysis. In addition, post-hoc comparisons were calculated to test the difference between treatment groups using a two-sample t-test.

For all analyses, the threshold was set at voxel-wise *p *< 0.001. To correct for multiple comparisons, we ran Monte Carlo simulations with AlphaSim to obtain corrected type I error http://afni.nimh.nih.gov/afni/doc/manual/AlphaSim. The results showed that, in our study, signal voxel threshold p < 0.001 combining 31 voxels has a corrected threshold of p < 0.05 at the cluster level. Thus, the threshold of voxel-wise p < 0.001 uncorrected with 31 contiguous voxels was used in this study.

## Results

Of seventy-seven healthy enrolled subjects, forty-eight (average age 26.4 ± 4.9; 24 males and 24 females) completed the study; each of the four treatment groups contained twelve subjects.

### MASS ratings

We used the MASS to quantify the intensity of subjects' sensations experienced during acupuncture treatment. Mean ratings differed between the genuine and sham group. For example, there were higher ratings of "dull pain", "throbbing pain" and "soreness" during genuine treatment, compared to sham. No sensations were rated higher in the sham group, compared to genuine. Additionally, there were sensations that did not differ between genuine and sham, e.g. "warm", "fullness", and "heaviness" yielded similar ratings in both treatment groups. To further assess whether there were differences in average MASS ratings amongst the four groups, we performed a fixed effect ANOVA using treatment mode (genuine and sham), and expectancy (high and low) as factors. The results showed that the four groups were significantly different, F (3,44) = 7.9, p = 0.0002. Subjects in both low and high expectancy genuine groups reported significantly greater MASS ratings than those in both sham groups (p < 0.002). There was no interaction between treatment mode and expectancy level (p = 0.8).

### fMRI results

**Figure 2:**
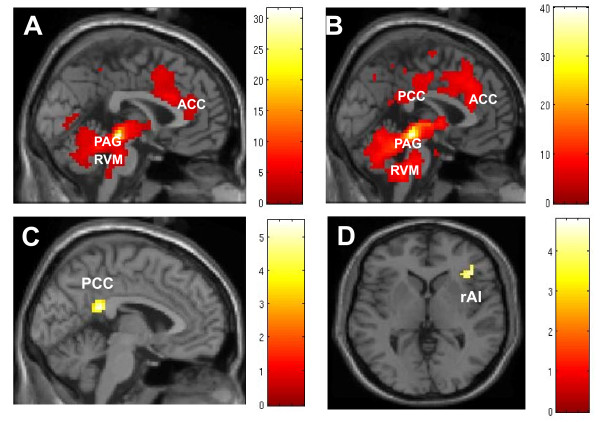
**Functional connectivity results**. A) Positive functional connectivity during sham treatment; B) Positive functional connectivity during genuine treatment. C) and D) Main effect of EA stimulation: Genuine > Sham (C) Sham > Genuine (D). The threshold was set to voxelwise *p *< 0.001 uncorrected with 31 contiguous voxels.

Functional connectivity results (as shown in Table 1 and Figure 2) demonstrated that during genuine EA, there was predominant positive functional connectivity between the PAG and nearby brain structures, including the bilateral PAG and surrounding areas (midbrain tegmentum, substantia nigra, raphe nucleus, hypothalamus, striatum, globus pallidum, left insula, thalamus, hippocampus, brain stem, and cerebellum). In addition, there was significant functional connectivity between the PAG and certain distant brain regions, including bilateral anterior cingulate cortex (ACC), medial prefrontal cortex (MPFC), middle cingulate cortex (MCC), posterior cingulate cortex (PCC), precuneus, inferior parietal lobule, and left postcentral gyrus.

**Table 1:** Functional connectivity results during genuine EA state and sham EA state

	Region	Z score	Number of voxels in cluster	Peak coordinate (x y z)
Genuine EA state	Bilateral PAG and surrounding areas (midbrain tegmentum, substantia nigra, raphe nucleus, hypothalamus, striatum, globus pallidum, left insula, thalamus, hippocampus, brain stem, cerebellum)	Inf	4992	-3 -27 -6
	ACC/MPFC, MCC and PCC	5.18	2269	3 21 45
	Right inferior parietal lobule	4.69	131	45 -39 33
	Right precuneus	4.06	42	12 -66 45
	Left precuneus	4.01	78	-9 -63 42
	Left inferior parietal lobule	3.95	42	-57 -39 30
	Bilateral medial prefrontal cortex	3.66	76	-6 -9 69
	Left inferior parietal lobule	3.65	41	-42 -54 51
	Left postcentral gyrus	3.6	32	-33 -21 39

Sham EA state	Bilateral PAG and surrounding areas (midbrain tegmentum, substantia nigra, raphe nucleus, hypothalamus, striatum, globus pallidum, left insula, thalamus, hippocampus, brain stem, cerebellum)	Inf	5989	0 -27 -9
	Right frontal operculum/inferior frontal gyrus	5.52		45 21 15
	Right anterior insula/inferior frontal gyrus	5.39		42 27 3
	Bilateral ACC/MPFC, MCC	5.44		0 35 27
	Left postcentral gyrus/inferior parietal lobule	5.45	146	63 -24 42
	Right inferior parietal lobule	5.37	72	-66 -33 33
	Bilateral PCC/precuneus	5.16	190	15 -36 42
	Right occipital gyrus	4.12	42	42 -81 27

Note: The threshold was set to voxel-wise p < 0.001 uncorrected with 31 continuous voxels. Peak coordinates refer to the MNI atlas

Similarly, functional connectivity results (as shown in Table 1 and Figure 2) during sham EA showed a positive functional connectivity between the PAG and nearby brain structures equivalent to those activated by genuine EA. Additional brain regions that exhibited a significant connection with the seed include the right frontal operculum, anterior insula, inferior frontal gyrus and occipital cortex.

The main effect of acupuncture mode, as indicated by comparison of genuine and sham EA groups (as shown in Table 2 and Figure 2), showed that: during genuine EA, the PAG showed more connectivity with the left PCC than during sham EA; and during sham EA there was more connectivity to the anterior right insula (rAI)/inferior frontal gyrus than during genuine EA. The main effect of expectancy, determined by direct comparison of HE and LE groups, showed no brain regions above the threshold. An interaction between the treatment mode and expectancy level was observed in the activation of the right superior parietal lobule.

**Table 2:** Functional connectivity differences between treatment expectancy levels and interaction (GEA: genuine EA; SEA: sham EA; HE: high expectancy; LE: low expectancy; GH: GEA paired with HE; GL: GEA paired with LE; SH: SEA paired with HE; SL: SEA paired with LE)

	Region	Z score	Number of voxels in cluster	Peak coordinate (x y z)
GEA > SEA	Left PCC	4.79	42	-6 -42 24
			
SEA > GEA	Right anterior insula/inferior frontal gyrus	3.94	62	33 27 -3

HE > LE	No region above the threshold			

LE > HE	No region above the threshold			
			
Interaction	Right superior parietal lobule	3.60	43	18 -51 57
			
GH > SH	No region above the threshold			
			
SH > GH	No region above the threshold			
			
GH > GL	Right inferior parietal lobule	4.32	36	39 -60 48
			
GL > GH	No region above the threshold			
			
SH > SL	No region above the threshold			
			
SL > SH	No region above the threshold			
			
GL > SL	No region above the threshold			
			
SL > GL	Right superior parietal lobule	4.20	47	21 -51 57

Note: The threshold was set to voxel-wise p < 0.001 uncorrected with 20 continuous voxels. Peak coordinates refer to the MNI atlas

Post-hoc comparisons were calculated between the following groups: genuine EA with high expectancy vs. sham EA with high expectancy, genuine EA with high expectancy vs. genuine EA with low expectancy, sham EA with high expectancy vs. sham EA with low expectancy, and genuine EA with low expectancy vs. sham EA with low expectancy. The results indicated that genuine EA with high expectancy showed more connectivity at the right inferior parietal lobule (39 -60 48, 36 voxles) compared with genuine EA with low expectancy. In addition, genuine EA with low expectancy showed less connectivity at the right superior parietal lobule (21 -51 57, 47 voxels) compared with sham EA with low expectancy. Other comparisons showed no brain regions above the threshold.

## Discussion

In this study, we investigated functional connectivity of the PAG during genuine and sham EA. We found that during both genuine and sham EA, the PAG was significantly connected with brain areas surrounding the PAG, including the midbrain tegmentum, substantia nigra, raphe nucleus, hypothalamus, striatum, globus pallidum, left insula, thalamus, hippocampus, brain stem, and cerebellum as well as distant regions such as ACC, MPFC, MCC, PCC and precuneus. However, genuine EA - relative to sham EA - showed significantly stronger connectivity between the PAG and left PCC, and significantly weaker connectivity between the PAG and rAI.

Our result that, during both genuine and sham EA, the PAG is functionally connected with surrounding regions and some distant regions, including the RVM and ACC, is similar to our previous study investigating the intrinsic functional connectivity of PAG during resting state using data from a different cohort [40]. Thus, neither genuine nor sham EA appeared to significantly disrupt the connectivity between PAG and the network of brain regions seen during resting state.

During genuine EA, we found that the PAG had a significantly stronger connectivity with the PCC. Through an examination of the existing literature, we could not find reliable evidence for a direct (monosynaptic) connection between the two structures; however, previous studies suggest a functional linkage between the PCC and brain stem [78]. A di-synaptic connection is the most parsimonious solution for which direct evidence exists. Thus, we speculate that this functional linkage may be conveyed via the thalamus and ACC, two regions that have direct connections with both PCC and PAG.

Previous studies have implicated the PCC's involvement in responses to treatment in chronic pain patients [79,80]. In a study by Niddam and colleagues [80], patients with myofascial pain syndrome were given painful stimulations during fMRI, and in between scanning sessions the same area was treated with low-intensity electrostimulation. When comparing responders and non-responders, a treatment effect was observed for responders in the dorsal midbrain, PCC, and the caudate [80].

In an early PET-study from 1995 [79], a normalization of attenuated PCC activity after non-opioidergic treatment was seen in patients with chronic neuropathic pain. Patients with localized peripheral neuropathic pain were treated with a regional nerve block using lidocaine, resulting in significant analgesic effects. Interestingly, the neural correlate to the pain alleviated state was an increase of cerebral blood flow in the ACC and PCC. Hseieh et al. suggest that the increased neural response in the PCC could reflect the altered subjective perception of pain relief rather than the afferent blockade. The PAG is one of the key regions in the descending pain inhibitory circuitry, enabling regulation of afferent pain signals. The strong connectivity between the PAG and the PCC in response to active treatment furthers the idea that the PCC plays an important role in pain treatment.

The PCC is a key region in the default mode network (DMN): a set of specific brain structures with intrinsic fluctuations that constitute a baseline of attention and wakefulness in the human brain [81-83]. In humans, the PCC has the highest level of resting cortical glucose metabolism [84], and is involved in processing intentions related to the self, self-awareness and conscious experience, which are key functions attributed to the DMN [78,85]. DMN activity has been shown to decrease in relation to task-evoked activity, and demonstrates an inverse relationship with the cognitive work load of the task [81-83]. Other studies show that activity in the DMN decreases in response to repeated painful stimuli [85], and chronic pain patients seem to exhibit permanently altered DMN activity. In a study by Baliki and colleagues [86], chronic low back pain patients displayed reduced activity in several key DMN regions compared with healthy subjects. We believe the results from this study indicate that EA may modulate the functional connectivity of the DMN, as evidenced by the increased connectivity of PCC with PAG during EA stimulation.

In this study we also found that during sham acupuncture, the PAG has stronger connectivity with the rAI compared with genuine acupuncture. The anterior insula is a key region in the pain matrix [87] and is involved in integration and interoception of pain [88-90] and pain modulation processes such as placebo analgesia [29,60]. In a more recent study, investigators found that the pre-stimulus functional connectivity between the PAG and the anterior insula can predict subsequent pain perception [91]. Thus, we speculate that EA stimulation may reduce brain responses to calibrated pain stimuli by interfering with the functional connectivity between the PAG and insula. Further research is needed to test this hypothesis.

An interaction between the acupuncture treatment modes and expectancy levels was observed in right superior parietal lobule. Studies have suggested that this region is involved in attention [92] and somatosensory perception modulation [93]. We speculate that our results may indicate that functional connectivity in genuine and sham EA are differentially modulated by expectancy levels, however further research is needed to fully understand the sources of observed functional connectivity in this study.

In this study, we did not find any significant functional connectivity changes between the high and low expectancy groups. We believed that this may be attributed to several reasons. 1) The PAG seed we chose for this study was identified in our previous analysis of the fMRI signal changes evoked by calibrated heat pain [30] as being selectively involved in mediating acupuncture treatment effects (genuine EA vs sham EA) and not expectancy effects (high expectancy vs low expectancy). 2) Although previous studies suggested that PAG is involved in expectancy evoked placebo analgesia [94-96] or attention modulation of pain [97], the involvement of PAG in these studies is observed during the pain application process; in contrast, this study measures functional connectivity changes during acupuncture treatment. Thus, our results are not necessarily in conflict with findings from previous studies. 3) The relatively small sample size may also prevent us from finding significant functional connectivity between the high and low expectancy conditions. Further study is needed to elucidate the influence of expectation of analgesia on the functional connectivity of PAG during the treatment phase.

## Conclusions

In summary, during continuous EA, functional connectivity changed significantly in brain regions including PCC and rAI. Our findings indicate the intrinsic functional connectivity changes among key brain regions in the pain matrix and default mode network during genuine EA compared with sham EA. We speculate that continuous genuine EA stimulation can modify the coupling of spontaneous activity in brain regions that play a role in modulating pain perception.

## Competing interests

The authors declare that they have no competing interests.

## Authors' contributions

CZ drafted the manuscript in conjunction with KJ, RL, AC, and JK; TJK, and RLG assisted with editing. RLG, TJK, and JK designed the experimental protocol; specifically, JK conceived of the functional connectivity study presented in this manuscript. JK and GP collected the data and coordinated the experiment, and were involved in data analysis with the assistance of CZ. PL and PT helped to design the fcMRI data analysis methods and performed the fcMRI data analysis. All authors read and approved the final manuscript.
